# The Effect of Corticosteroid Therapy on Choroidal Thickness in Patients With Covid-19 Infection: A Prospective, Comparative, and Observational Study

**DOI:** 10.7759/cureus.32835

**Published:** 2022-12-22

**Authors:** Hatice Kubra Sonmez, Cem Evereklioglu, Zeynep Ture Yuce, Duygu G Sevim, Ozge Temizyurek, Osman Ahmet Polat, Fatih Horozoglu

**Affiliations:** 1 Department of Ophthalmology, Erciyes University Medical Faculty, Kayseri, TUR; 2 Department of Clinical Microbiology and Infection, Erciyes University Medical Faculty, Kayseri, TUR

**Keywords:** steroid therapy, choroidal thickness, coronavirus disease, covid-19, sars-cov-2

## Abstract

Background: Coronavirus disease 2019 (Covid-19) has many different ocular manifestations. This study evaluates the effects of the disease and the steroid used in this disease on ocular structures.

Purpose: To evaluate the effects of Covid-19 and the steroids used in the treatment of severe infection on ocular structures and choroidal thickness.

Methods: This prospective study included 76 eyes of 76 patients who were hospitalized due to Covid-19 and 30 eyes of 30 healthy volunteering controls. Group I included 35 eyes who were hospitalized due to moderate-to-severe involvement that received steroid treatment, group II included 41 eyes with moderate involvement that did not require steroid treatment, and group III included 30 eyes with age- and gender-matched control subjects. Ophthalmological examination and imaging results of the patients obtained in the third week and third month after the diagnosis were compared between the groups.

Results: Mean age of all participants was 40.2 ± 6.1 years. In the third week after the diagnosis of Covid-19, choroidal thickness in all regions (subfoveal, nasal, and temporal) was significantly greater in group I than in group II (for all, *p*<0.001). Moreover, choroidal thicknesses were significantly higher in group I and group II than in the control group (for all, *p*<0.001). In the third month, all the groups had similar choroidal thickness values (for subfoveal, nasal, and temporal; *p*=0.058, *p*=0.111, *p*=0.079, respectively).

Conclusion: Our findings showed that Covid-19 infection causes choroidal thickening by affecting the choroidal layer and that steroid treatment further increases this thickness in the acute period. In addition, the reversal of this thickening to the normal level within a period of three months indicates that the effect of the disease on the choroid is reversible.

## Introduction

Coronavirus disease 2019 (Covid-19), which manifested in Wuhan, China with severe pneumonia in December 2019, entered our lives with the declaration of a pandemic by the World Health Organization (WHO) in January 2020 [1]. In addition to its severe effects on the respiratory system, the virus has also been found to have numerous involvements outside the respiratory system, with the most commonly involved organs and tissues including the cardiac system, kidneys, central nervous system, gastrointestinal tract, and eye. Additionally, thrombotic complications resulting from direct effects of the virus or the endothelial dysfunction caused by the virus have also been shown to cause extrapulmonary involvement [2,3].

Ocular manifestations caused by Covid-19 have been reported such as episcleritis, conjunctivitis, keratitis, foreign body sensation, dry eye, epiphora or retinal vein occlusion due to thrombotic complications, and paracentral acute middle maculopathy (PAMM) [4-6]. Ocular involvement has been associated either directly with viral interaction or indirectly with the inflammatory response caused by the virus. Though rarer than ocular surface involvements, posterior segment involvements have also been reported, namely including acute macular neuroretinopathy, retinal vein occlusion, PAMM, intraretinal hemorrhages, and cotton-wool spots [7]. Additionally, in some case series, ocular involvement like choroidopathy has also been reported [8]. In a confirmatory manner, some other studies detected increased choroidal thickness in choroidal measurements performed after the diagnosis of Covid-19 [9,10].

Studies have shown that the use of systemic steroids in Covid-19 patients with severe respiratory involvement and severe hypoxemia has a positive effect on the prognosis of the disease [11]. Some other studies have reported that systemic steroids given even for a short period of time pose a risk in terms of ocular side effects, with the most common side effects including posterior subcapsular cataract, choroidal/retinal embolism, and increased intraocular pressure [12]. Moreover, it is commonly reported that systemic steroid use, particularly at high doses, causes an increase in choroidal thickness and choroidal vascular permeability, leading to diseases such as central serous chorioretinopathy (CSCR) [13].

In this study, we aimed to evaluate the effects of corticosteroid usage on Covid-19 infection on anterior and posterior segments of the eye and evaluated the choroidal thickness.

## Materials and methods

The study was designed as a prospective, comparative, observational case-control study and included 76 eyes of 76 patients aged 18-45 years who were hospitalized due to severe acute respiratory syndrome coronavirus 2 (SARS-CoV-2) at Erciyes University Department of Ophthalmology and 30 eyes of 30 healthy volunteering controls. Ophthalmological examination and imaging results of the patients were obtained in the third week and third month after the diagnosis of Covid-19 infection and were compared with the results of the control group. The study was approved by the local ethics committee (Approval Number: 2021/252). Informed consent was obtained from each participant.

Study groups and inclusion/exclusion criteria

Group I included 35 eyes of 35 patients who were hospitalized due to moderate-to-severe pulmonary involvement that did not require intubation and received steroid treatment (methylprednisolone (MP)). The oxygen saturation (SpO2) of the patients in Group I was in the range of 89-93%, and all of them received oxygen support with a high-flow nasal cannula or mask. Group II included 41 eyes of 41 patients with moderate involvement that did not require steroid treatment, and group III included 30 eyes of 30 healthy age- and gender-matched volunteers. Patients in group I had completed their total steroid course prior to the study. All the patients received low-molecular-weight heparin (LMWH) therapy.

Patients with systemic diseases such as diabetes and hypertension, history of ocular surgery, trauma, glaucoma, uveitis, retinal pathologies, smoking, cataracts other than posterior subcapsular cataracts, a spherical equivalent of more than 3.00 diopters, history of systemic steroid therapy due to other reasons, hospitalization periods longer than three weeks, history of chronic radiation exposure, low signal strength (with a signal quality of 8 and below) on optical coherence tomography (OCT), and a history of medical treatment other than LMWH treatment (e.g. antibiotics, antimalarial drug) were excluded from the study.

Data collection

For standardization purposes, the right eye of patients and controls were included in the study. Ophthalmological examination (best corrected visual acuity (BCVA), slit lamp examination (biomicroscopy), fundoscopy, intraocular pressure measurement (Goldmann applanation tonometry), and autorefractometry (TonoRef II, Nidek, Japan)) was performed at the third week and third month after the diagnosis of Covid-19. BCVA was assessed using the Snellen decimal scale converted to a logarithm of the minimum angle of resolution (logMAR). Fundus examination was performed using a +90 D lens after pupillary dilation. The Lens Opacities Classification System III (LOCS III) was used for grading cataract severity [14]. In all groups, choroidal thickness was measured with enhanced depth image (EDI) on spectral-domain OCT (SD-OCT, Heidelberg Engineering Inc., Software version 6.3.3.0, Heidelberg, Germany). In the control group, the same examinations and measurements were performed only once.

**Figure 1:**
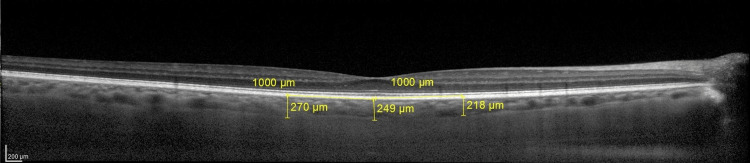
Enhanced depth imaging (EDI) mode horizontal spectral-domain optic-coherence tomography image of the macula region. Choroidal thickness measurements were obtained from subfoveal, nasal and temporal measurements were obtained from 1000 μm distance to the fovea.

Subfoveal choroidal thickness (SFCT) and the choroidal thicknesses at 1000 μm nasal (N1000) and 1000 μm temporal (T1000) to the fovea were measured manually from the outer portion of the reflective line corresponding to the inner scleral border with the built-in caliper of the SD-OCT device software. The choroidal thickness measurement procedure is shown in Figure 1. All the OCT measurements were performed by the same operator between 9 and 11 AM to prevent diurnal changes.

Interobserver evaluations

All the ophthalmological examinations and measurements were evaluated separately by two ophthalmologists (HKS, ÖT), and patients with similar results were included in the study.

Statistical analysis

Data were analyzed using Statistical Product and Service Solutions (SPSS) (IBM SPSS Statistics for Windows, Version 26.0, Armonk, NY). The normal distribution of continuous variables was assessed using the Kolmogorov-Smirnov test and Histogram plots. Based on these analyses, continuous variables showing normal distribution were expressed as mean ± standard deviation (SD) and those with nonnormal distribution were expressed as median (1st-3rd quartile). Categorical variables were expressed as percentages (%). Continuous variables with normal distribution were compared among three or more independent groups using the One-Way ANOVA test and the continuous variables with nonnormal distribution were compared using the Kruskal-Wallis test. The Bonferroni test was used as a post hoc test to determine the different groups in One-Way ANOVA. In pairwise comparisons, continuous variables with normal distribution were compared using paired-samples t-test and the continuous variables with nonnormal distribution were compared using the Wilcoxon signed-rank test. Statistical graphs were created using GraphPad Prism Version 8 (GraphPad Corp, San Diego, USA). A p-value of <0.05 was considered significant.

## Results

The mean age of all participants was 40.2 ± 6.1 years. The groups were similar in terms of female-to-male ratio, mean age, mean body mass index (BMI), and mean BCVA. Table 1 presents the demographic and clinical characteristics of the participants. The mean total dose of MP administered in group I was 852 ± 226 mg.

No significant difference was found between the patient groups and the control group with regard to mean intraocular pressure measured at the post-Covid-19 third week and third month (p>0.05).

Posterior subcapsular cataract (PSC) developed in one patient in group I at the third month. This patient had cataract grade 1 (P1) according to LOCS III and had deterioration in vision in one line of Snellen acuity. No cataract was detected in any participant in groups II and III.

At the third week after the diagnosis of Covid-19, choroidal thickness in all regions (subfoveal, nasal, and temporal) was significantly greater in group I than in group II (for each, p<0.001). Moreover, choroidal thickness was significantly higher in group I and group II than in the control group (for each, p<0.05).

At the third month after the diagnosis of Covid-19, all the groups had similar choroidal thickness values (subfoveal, nasal, and temporal; p=0.058, p=0.111, p=0.079, respectively). A comparison of the third-week and third-month choroidal thickness values of the groups is presented in Table 1.

**Table 1:** Demographic, clinical characteristics, and comparative choroidal thickness results of the groups. BMI: body mass index, BCVA: best corrected visual acuity, IOP: intraocular pressure, SI-PSC: steroid-induced posterior subcapsular cataract A p-value less than 0.05 indicates a statistically significant difference. *All three groups are statistically different from each other and p<0.001 for all.

	Group 1 (n=35)	Group 2 (n=41)	Controls (n=30)	p-value
Age (years)	41.3 ± 6.1	38.2 ± 7.9	40.9 ± 5.4	0.122
BMI (kg/m^2^)	28.1 ± 5.1	27.2 ± 4.6	27.6 ± 4.8	0.451
Gender (Female/Male)	14/21 (40/60%)	15/26 (36/64%)	13/17 (43/57%)	0.321
BCVA (LogMAR)	0.09	0	0	0.281
Mean IOP (mmHg) - 3^rd^-week	12.71 ± 1.96	13.90 ± 2.13	12.80 ± 2.31	0.196
Mean IOP (mmHg) - 3^rd^-month	12.86 ± 1.97	14.0 ± 2.11		0.182
SFCT (µm) - 3^rd^-week	274.31 ± 37.89	243.73 ± 28.50	229.67 ± 26.02	<0.001*
SFCT (µm) - 3^rd^-month	238.80 ± 27.09	224.02 ± 24.03		0.058
N1000 (µm) - 3^rd^-week	249.54 ± 46.09	224.76 ± 31.57	208.40 ± 19.09	<0.001*
N1000 (µm) - 3^rd^-month	221.97 ± 29.38	216.46 ± 26.70		0.111
T1000 (µm) - 3^rd^-week	264.26 ± 40.41	238.29 ± 27.38	213.30 ± 18.20	<0.001*
T1000 (µm) - 3^rd^-month	219.71 ± 12.63	222.24 ± 18.10		0.079
SI-PSC - 3^rd^-week	0/35	0/41	0/30	N/A
SI-PSC - 3^rd^-month	1/35	0/41		

**Figure 2:**
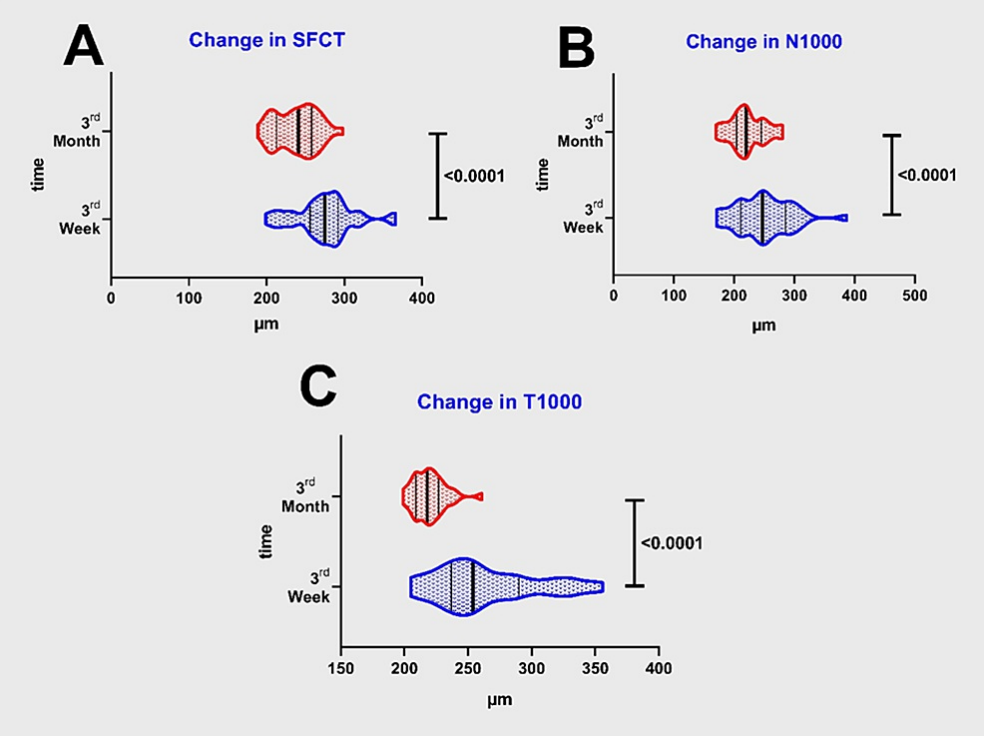
Changes in choroidal thickness in group 1, at third week and third month. A - Change in subfoveal choroidal thicknesses (SFCT). B - Change in nasal (N1000) choroidal thicknesses. C - Change in temporal (T1000) choroidal thicknesses.

**Figure 3:**
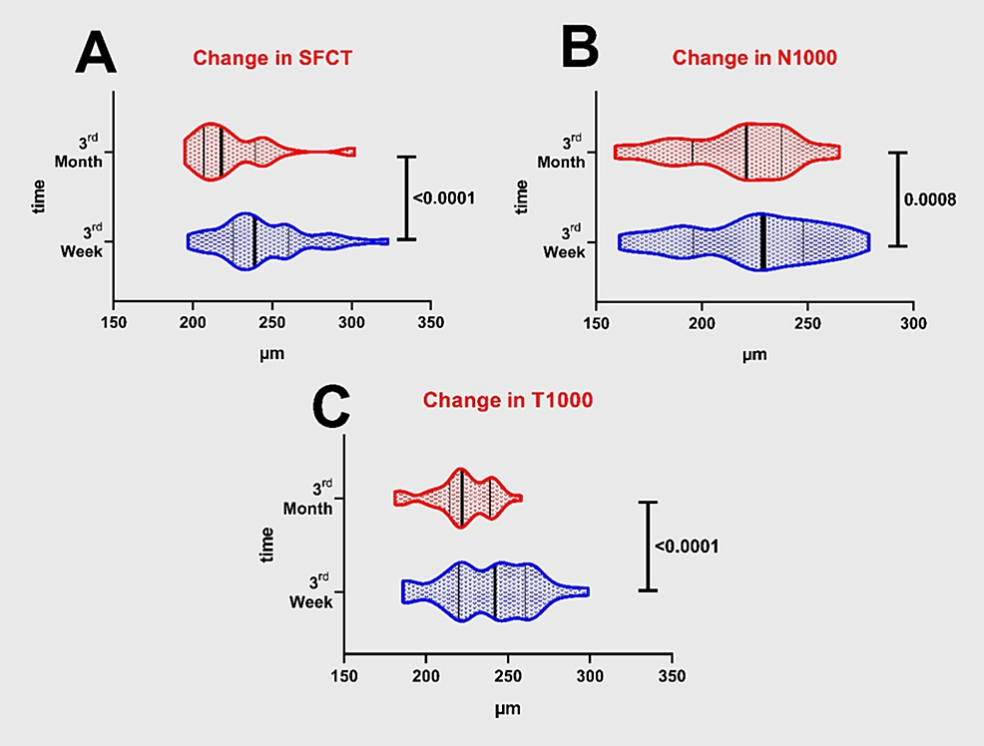
Changes in choroidal thickness in group 2, at third week and third month. A - Change in subfoveal choroidal thicknesses (SFCT). B - Change in nasal (N1000) choroidal thicknesses. C - Change in temporal (T1000) choroidal thicknesses.

In contrast, a comparison of the patient groups with regard to the change between the third-week and third-month measurements revealed a significant decrease in choroidal thickness in all regions (for group I; p<0.0001 for each and for group II; subfoveal p<0.0001, nasal p=0.0008, temporal p<0.0001). Figure 2 and Figure 3 present the changes in the choroidal thickness values of the groups during the nine-week period between the third-week and the third-month measurements.

## Discussion

Systemic ocular manifestations of Covid-19 have been reported in numerous studies [4-9]. In contrast, the present study evaluated the role of steroid use in the development of these manifestations and also examined the effects of Covid-19 on ocular structures and choroidal thickness. Our results showed that Covid-19 leads to an increase in choroidal thickness in young patients during the early stage of the disease and that such an increase is exacerbated by steroid management. However, a comparison with the control group indicated that the increase in choroidal thickness was temporary and that the choroidal thickness returned to normal at the third month after the diagnosis of Covid-19.

Abrishami et al. compared a total of 30 steroid-free Covid-19 patients with mild and moderate involvement with healthy controls and detected choroidal vascular dilatation in 68% of the patients [15]. The researchers showed increased SFCT in the patient group compared to the control group and also detected hyper-reflective dots in different retinal layers as well as pigment epitheliopathy findings, albeit at a low rate, in SD-OCT images in the patient group. In a previous OCT angiography study, Turker et al. found an increase in choriocapillaris flow along with a decrease in vascular flow in the retinal capillary plexuses in Covid-19 patients [16]. Another study evaluated early and late choroidal thickness and choroidal vascularity index in Covid-19 patients and found that the choroidal changes detected in the early period resolved at the nine months postinfectious period [17]. Abdelmassih et al. evaluated OCT images in Covid-19 patients and obtained findings in favor of choroidopathy in indocyanine green (ICG) angiography as well as choroidal thickening [8]. Another study reported that the choroidal vascularity index increased in the first month after the diagnosis of Covid-19 and the authors suggested that this result was due to the development of inflammation [18]. Gundogan et al. found an increase in the choroidal thickness and in the thickness of retinal vessels in severe Covid-19 patients during the acute period [10]. Similarly, in our study, an increase in choroidal thickness was also observed in the acute period. However, our findings further showed that this increase lasted for a maximum of three months and was reversible. This finding is consistent with the findings of some studies that showed a decrease in choroidal thickness toward the baseline level during the recovery period. Based on our findings, we consider that the severe systemic inflammation observed in our patients with severe disease activity had an effect on the choroidal layer, thus causing an increased stromal thickening.

It is known that hypoxia may also cause changes in choroidal blood flow. Hayreh, SS showed that hypoxia can cause an increase in choroidal blood flow by vasodilation [19]. Türker et al. reported an increase in choroidal perfusion in Covid-19 patients. They linked it to hypoxia-induced vasodilation in the choroidal vessels and an increase in vascular flow in the choroid as a result of systemic inflammation [16]. In our study, hypoxia may have affected choroidal blood flow. However, the fact that the first measurements were performed after the patients received oxygen therapy may have minimized the effect.

It is commonly reported that one of the many ocular side effects of corticosteroids is undoubtedly seen on the retina and choroid [13,20-22]. In a multicentric study including 538 eyes with CSCR, it was reported that patients that used corticosteroids for more than three months had increased choroidal thickness and choroidal vascular permeability as well as a higher rate of retinal pigment epithelium (RPE) dysfunction [13]. Another study reported that there was a greater increase in choroidal thickness in the steroid-using group compared to the idiopathic group in patients with acute CSCR, while there was no significant difference between the two groups with regard to choroidal vessel thickness [20]. Some studies have shown that systemic corticosteroids affect the blood-retina barrier (BRB) and cause increased choriocapillaris permeability and that systemic corticosteroids are also a risk factor for choroidal vascular hyperpermeability [21,22]. On the other hand, studies on the choroid in long-term use of high-dose corticosteroids have shown a decrease in the choroidal vascularity index, vasoconstriction in the choroidal vessels, and consequently a decrease in choroidal thickness [23,24]. In our study, in line with the literature, the choroidal thickness of Covid-19 patients was greater in the steroid-using group compared to the steroid-free group. We consider that the increased choroidal thickness observed in the steroid-free group was mostly due to inflammation and that the disease resulted in an increase in vascular permeability by causing choroidal vasculature endothelial dysfunction. On the other hand, although steroids are considered to reduce choroidal and systemic inflammation, our findings indicated that thickening in the steroid-using group reversed within the same period as that of the steroid-free group. Accordingly, these findings implicate that although the treatment only affects the thickness due to the inflammation in steroid-using patients, a certain period of time is required for vascular hyperpermeability and the repair of the impaired BRB. In addition, the greater reduction in choroidal thickness in the steroid-using group may also be due to the vasoconstriction effect on the choroidal vessels caused by corticosteroids in the long term.

Although the mechanism of cataract induction remains unclear for steroid-induced posterior subcapsular cataract (SI-PSC), it is considered to be caused by glucocorticoid-inducible gene expression in lens epithelial cells and other intraocular cells [25]. In a study including 37 patients, it was shown that SI-PSC development started in a period ranging from one to six months after steroid use [26]. In a study conducted on patients receiving long-term glucocorticoid therapy, the mean time from the onset of steroid use to the onset of cataract development was found to be 6.5 ± 3.6 years [27]. To our knowledge, cataract development associated with Covid-19 has not been reported in the literature. In our study, only one patient who received steroids had the onset of SI-PSC in the third month after the diagnosis of Covid-19. This finding, in line with the literature, implies that short-term and low-dose steroid treatment does not cause serious cataract development in Covid-19 patients. On the other hand, in one patient that developed cataracts, we believe that cataracts could be classified as a dose-independent idiosyncratic effect.

Our study was limited in several ways. First, it had a small number of cases and a short follow-up period. Second, the differences in disease severity and oxygen saturation are likely to have affected the choroidal thickness in both patient groups, albeit minimally. In addition, no baseline examinations and measurements were performed at the time of diagnosis in any patient due to technical difficulties and the risk of infection transmission during the pandemic period. A control group was included in the study in order to alleviate these limitations to some extent.

## Conclusions

Our findings indicated that although there is an increase in choroidal thickness in patients with Covid-19 during the early stage of the disease, this increase is further exacerbated with steroid treatment but it is often reversible. In addition, our results showed that the steroids administered to support treatment did not have a significant role in the development of cataracts. There are studies in the literature on ocular complications and structural choroidal changes induced by Covid-19. However, to our knowledge, our study is the first of its kind to investigate the role of steroid treatment in such patients. Considering the clinical features of retinal vasculopathy and choroidopathy associated with Covid-19, we believe that patients requiring steroid treatment have a higher risk of choroidal disorders and may require closer ophthalmological follow-up. Further studies with larger series and longer follow-up periods including the examination findings of the patients at the time of diagnosis are needed to substantiate our findings.
